# Comparison of the Chromosome Banding Pattern in the 2*n* = 56 Cytotypes of *Nannospalax leucodon* and *N. xanthodon* from Turkey

**DOI:** 10.1155/2014/121690

**Published:** 2014-01-23

**Authors:** Atilla Arslan, Apdil Arısoy, Jan Zima

**Affiliations:** ^1^Department of Biology, Faculty of Science, Selçuk University, 42031 Konya, Turkey; ^2^Institute of Vertebrate Biology, Academy of Sciences of the Czech Republic, 603 65 Brno, Czech Republic

## Abstract

We present the karyotype characteristics of five cytotypes of mole rats (*Nannospalax*) with 56 chromosomes revealed by the C-banding and AgNOR staining analyses. We attempt to investigate if the specific distinction between the populations from Thrace (*N. leucodon*) and Anatolia (*N. xanthodon*) is reflected also in their karyotypic differentiation. The specimens from each of the five populations studied revealed a distinct karyotype which was different from those found in other populations. The fundamental number of autosomal arms varied from 68 to 72. The amount of C-heterochromatin was larger in the Thrace sample of *N. leucodon* compared to the Anatolian population of *N. xanthodon*. The active NOR sites were recorded on five autosomal pairs in *N. leucodon*, whereas only three or four pairs bearing NOR were observed in *N. xanthodon*. Differences between the studied populations were quantified in the analysis of the distribution pattern of the C-positive bands and the AgNOR sites in individual chromosomes which indicated a basal position of the Thrace sample of *N. leucodon* and its divergence from other studied populations. The karyotypes of the 56-chromosome populations of *N. leucodon* and *N. xanthodon* are thus distinctly different.

## 1. Introduction

The mole rats (Muridae, Spalacinae) [1] represent an attractive model for various biological and evolutionary studies [2, 3]. The attraction of mole rats is due to several reasons, related particularly to the unusual subterranean way of life and various unique adaptive features in their behaviour, sensory biology, and population ecology. Another remarkable character of mole rats is their exceptionally extensive karyotypic variation which has been recorded between populations. Many geographic populations were shown to possess a distinct chromosomal complement differing from other populations. A population or a group of populations with a characteristic karyotype is commonly called the cytotype or the chromosomal race. Some authors [4] proposed that individual cytotypes may be recognized as presumptively good biological species.

The amazing chromosomal variation in the mole rats is not reflected in the extent of morphological differentiation. The external phenotype of mole rats is quite uniform as a consequence of selection constraints resulting apparently from their strictly underground habits. The morphological uniformity is obviously responsible for frequently changing and complicated systematics of this group. Currently, the mole rats are classified in two distinct genera, *Spalax* and *Nannospalax* [5]. The extensive intraspecific variation in the karyotype is typical for the latter genus which is usually divided into three species with nearly parapatric distribution. Southeastern Europe is inhabited by *N. leucodon*, Anatolia and Transcaucasia by *N. xanthodon*, and other parts of the Middle East and northeastern edges of Africa by *N. ehrenbergi *[1, 6, 7]. The borders between the distribution ranges of individual species are not clear in details and their morphological discrimination is difficult.

Turkey can be considered a core area of differentiation processes in chromosomal evolution within mole rats [8–10] and all the three *Nannospalax* species were recorded in this country. *N. leucodon* is presumably restricted to the European part of the country called Thrace and its occurrence in the western parts of Anatolia (the Asiatic part of Turkey) is not sure [7, 11]. The karyotype of Turkish populations of *N. leucodon *comprises 56 chromosomes (e.g., [10, 12, 13]), but the same diploid number was recorded also in various populations of *N. xanthodon* from Anatolia (e.g., [12–16]). In these studies the conventionally chromosomes were mostly studied and the banding data are largely lacking.

In the present study we aim to improve karyotypic characteristics of various cytotypes with 56 chromosomes included either in *N. leucodon* or *N. xanthodon *by the C-banding and AgNOR staining analyses. We attempt to investigate if the specific distinction between the populations from Thrace and Anatolia is reflected also in their karyotypic differentiation. To answer this question, we compare the results with other reports of chromosomal banding patterns found in Turkish populations of *Nannospalax* [17–22].

## 2. Material and Methods

**Figure 1:**
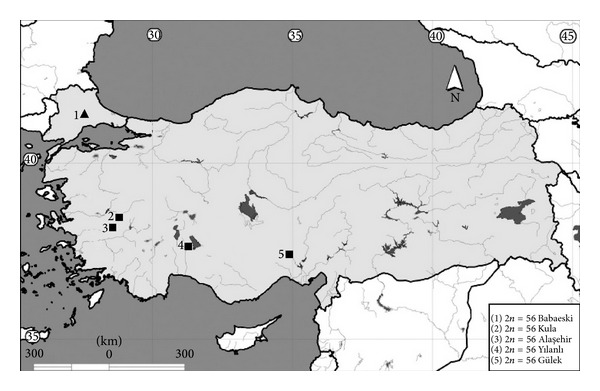
Collecting sites of *Nannospalax leucodon* (▲) and *N. xanthodon* (■) in Turkey. The numbering of sampling localities corresponds to data in Table 1.

Cytogenetic analyses were performed in 15 specimens of *N. leucodon *and *N. xanthodon *from five Turkish populations. The specimens were caught with a metal pipe-type trap [23]. This trapping method enables to obtain living individuals without any injury. The number of specimens analyzed and location of the collection sites of mole rats are shown in Figure 1 and Table 1. The study was done and the specimens were obtained with the permission of Republic of Turkey, Ministry of Forest and Water Works (Permission no. 7468).

Standard voucher specimens (skins and skulls) are deposited at Selçuk University, Biology Department, Faculty of Science, Konya, Turkey.

Karyotype preparations were obtained in the field from bone marrow after colchicine treatment [24]. Air-dried preparations were stained conventionally by Giemsa. Constitutive heterochromatin and nucleolus organizer regions (NORs) were detected by the techniques of C-banding [25] and silver staining of nucleolar organizer regions [26], respectively. From each specimen, 10 to 20 slides were prepared, and at least 20 well-spread metaphase plates were analysed. The system of classification of chromosomes according to the centromere position was adopted after Hsu and Benirschke [27], and the biarmed (metacentric, M, submetacentric, SM, subtelocentric, ST) and uniarmed (acrocentric, A) chromosomes were distinguished. The fundamental number of autosomal arms (NFa) and the number of all chromosomal arms in the female complement (NF) were calculated. We followed the arrangement of chromosomes in the karyotype applied in some previous papers [17, 18, 20]. The two large acrocentric autosomal pairs which can be reliably recognized were arranged as the first and second pairs in the complement. The other biarmed and acrocentric autosomes were arranged according to their size, respectively.

The distribution of the C-positive bands and AgNOR sites on individual chromosomes was summarized in the presence/absence matrix and a Neighbour Joining Clustering analysis was performed based on the character dataset using the PAST programme [28].

## 3. Results

### 3.1. Thrace, Kırklareli Province, Babaeski Population

**Figure 2:**
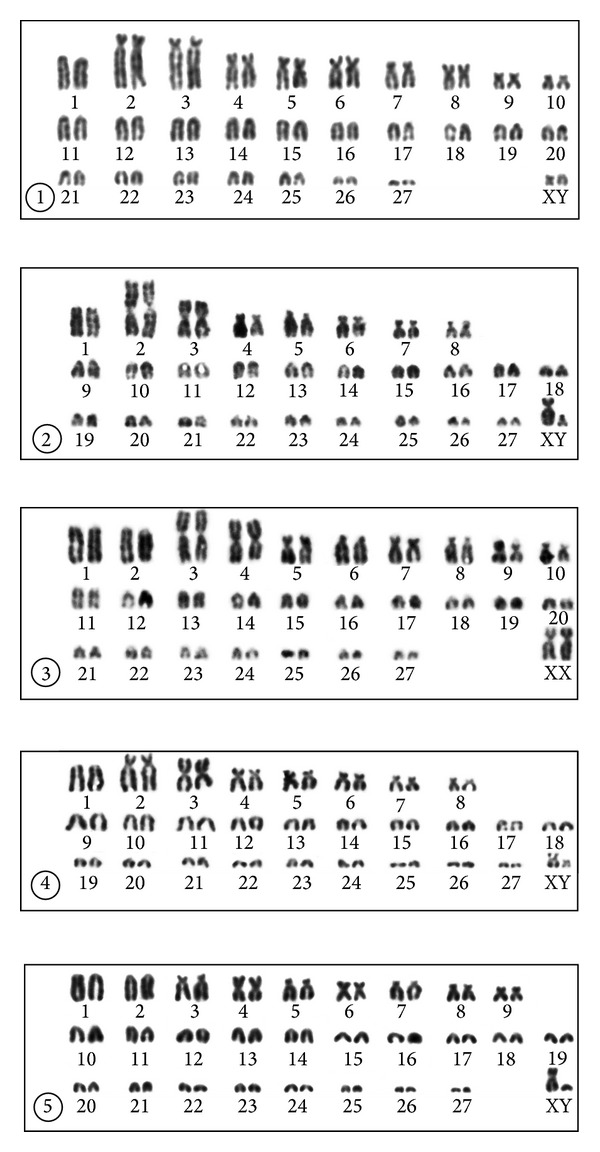
Standard karyotypes of specimens of *Nannospalax leucodon* from Babaeski (1) and *N. xanthodon* from Kula (2), Alaşehir (3), Yılanlı (4), and Gülek (5).

**Figure 3:**
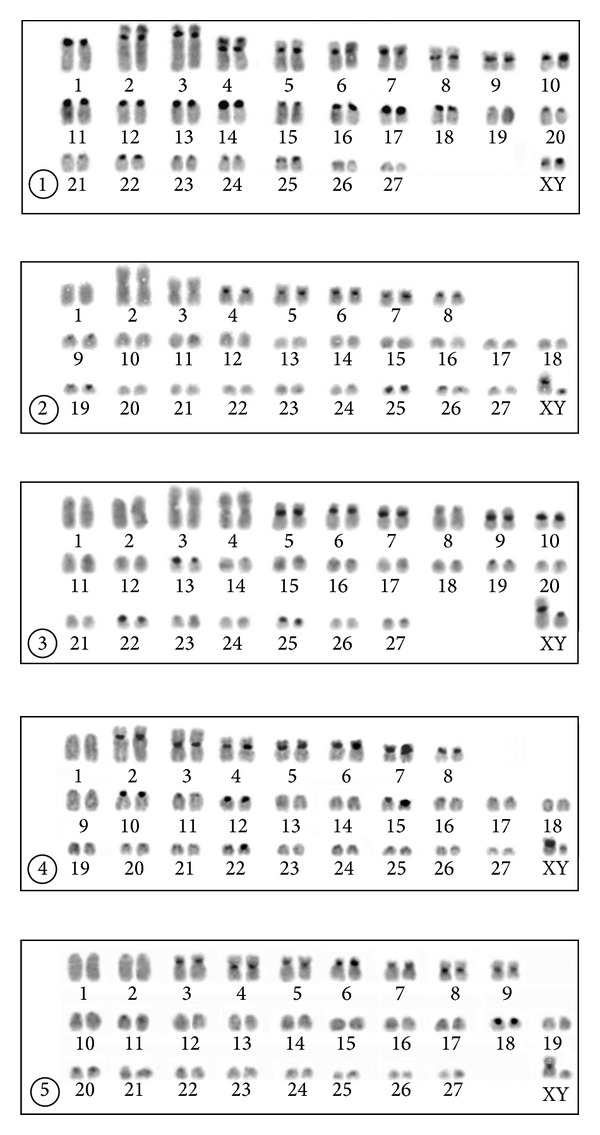
C-banded karyotypes of specimens of *Nannospalax leucodon* from Babaeski (1) and *N. xanthodon* from Kula (2), Alaşehir (3), Yılanlı (4), and Gülek (5).

**Figure 4:**
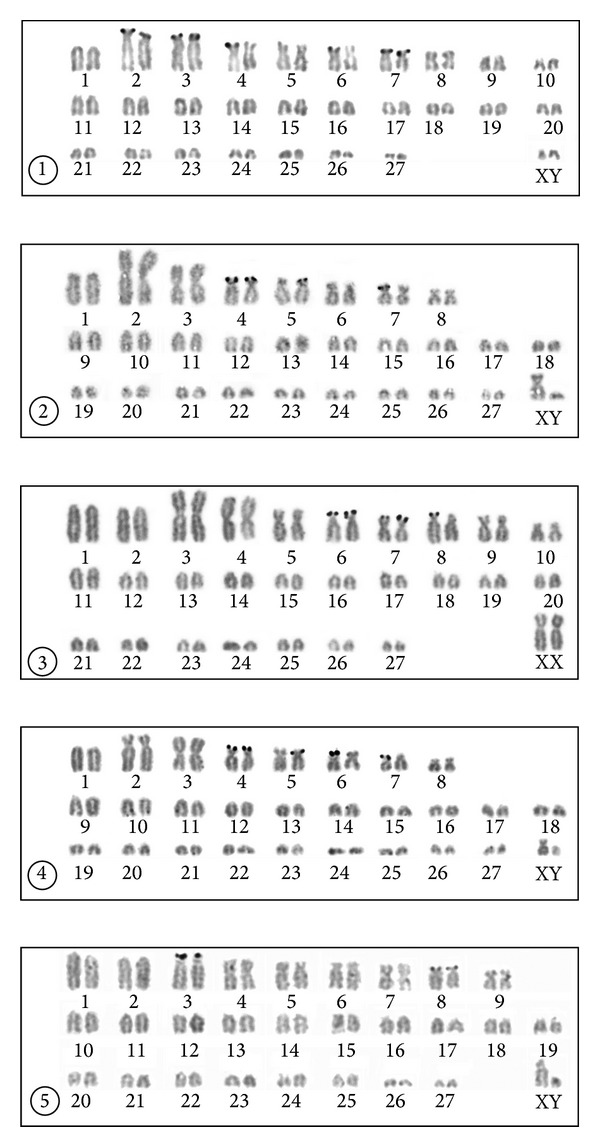
Silver stained karyotypes of specimens of *Nannospalax leucodon* from Babaeski (1) and *N. xanthodon* from Kula (2), Alaşehir (3), Yılanlı (4), and Gülek (5).

The karyotype of a male and a female consisted of 56 chromosomes including a single large acrocentric (no. 1), two large subtelocentric (2, 3), seven medium-sized submetacentric or subtelocentric (4–10), and 16 acrocentric autosomal pairs of gradually diminishing size (11–27) (NFa = 72). The X chromosome was a small metacentric, and the Y chromosome a small acrocentric (NF = 76) (Figure 2(1)). The dark C-bands were observed in pericentromeric areas of all the biarmed and most of the acrocentric autosomes. Three acrocentric autosomal pairs were C-negatively stained (nos. 19, 26, and 27). Slight telomeric dark C-bands were observed in the short arms of the three largest biarmed autosomal pairs (2, 3, and 4). The X chromosome had a centromeric C-positive area and the Y chromosome possessed a distinct dark pericentromeric C-positive band extending over the proxima third of the chromosome (Figure 3(1)). The NORs were observed in the telomeric regions of the short arms of the autosomes 2, 3, 4, 6, and 7. In some cells, only one homologue of the pairs 4 and 6 bore the positive silver signal (Figure 4(1)).

### 3.2. Anatolia, Manisa Province, Kula Population

The karyotype of the two males consisted of 56 chromosomes including a large acrocentric (no. 1), a large and a medium-sized metacentric (2, 3), five medium-sized submetacentric or subtelocentric (4–8), and 19 acrocentric autosomal pairs of gradually diminishing size (nos. 9–27) (NFa = 68). The X chromosome was a large submetacentric, and the Y a small acrocentric (NF = 72) (Figure 2(2)). The dark C-bands were observed in pericentromeric areas of five biarmed (4–8) and four acrocentric autosomes (9, 19, 25, and 26). The X and Y chromosomes had distinct centromeric C-positive bands (Figure 3(2)). The NORs were observed in the telomeric regions of the short arms of the autosomes nos. 4, 5, and 7. In the pairs 5 and 7 the positive signal was observed in only one homologue of the pair in some cells (Figure 4(2)).

### 3.3. Anatolia, Manisa Province, Alaşehir Population

The karyotype of the five specimens examined consisted of 56 chromosomes including two large acrocentric (nos. 1, 2), two large metacentric (3, 4), six medium-sized to small submetacentric or subtelocentric (5–10), and 17 acrocentric autosomal pairs of gradually diminishing size (11–27) (NFa = 70). The X chromosome was a large submetacentric, and the Y chromosome was a small acrocentric (NF = 74) (Figure 2(3)). The dark C-bands were observed in the pericentromeric areas of five biarmed (5–7, 9, and 10) and four acrocentric autosomes (13, 19, 22, 25). The sex chromosomes had dark centromeric C-positive bands (Figure 3(3)). The NORs were observed in the telomeric regions of the short arms of the autosomes 6, 7, and 8, with an occasionally heterozygous signal on the autosome 8 (Figure 4(3)).

### 3.4. Anatolia, Isparta Province, Yılanlı Population

The karyotype of the three males consisted of 56 chromosomes including a large acrocentric (no. 1), a large submetacentric and a large metacentric (2, 3), five submetacentric or subtelocentric (4–8), and 19 acrocentric autosomal pairs of gradually diminishing size (9–27) (NFa = 68). The X and Y chromosomes were small submetacentrics (NF = 72) (Figure 2(4)). The dark C-bands were observed in the pericentromeric areas of seven biarmed (2–8) and four acrocentric autosomes (10, 12, 15, and 22). The X chromosome has a distinct pericentromeric C-positive band and the Y chromosome has a slight centromeric C-positive band (Figure 3(4)). The NORs were observed in the telomeric regions of the short arms of the autosomes 4–7, with an occasionally heterozygous signal on the autosomes 5, 6, and 7 (Figure 4(4)).

### 3.5. Anatolia, Mersin Province, Gülek Population

The karyotype of the three males consisted of 56 chromosomes including two large acrocentric (nos. 1, 2), a metacentric (4), six submetacentric or subtelocentric (3, 5–9), and 18 acrocentric autosomal pairs of gradually diminishing size (10–27) (NFa = 68). The X chromosome was a medium-sized submetacentric, and the Y chromosome a small acrocentric (NF = 72) (Figure 2(5)). The dark C-bands were observed in the pericentromeric areas of seven biarmed (3–9) and a single acrocentric autosomal pair (18). The X chromosome had a pericentromeric C-positive band and the Y chromosome was entirely C-negative (Figure 3(5)). The NORs were observed in the telomeric regions of the short arms of the subtelocentric autosomes nos. 3 and 8 (Figure 4(5)).

### 3.6. Karyotypic Relationships between the Populations

**Figure 5:**
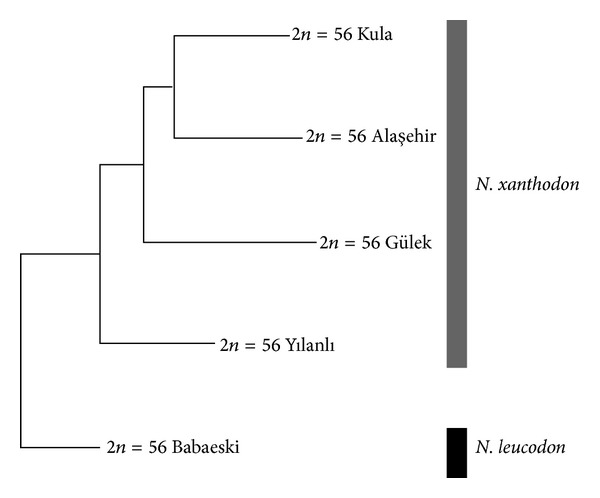
The neighbour-joining tree of the relationships among the studied populations based on the distribution of the C-positive bands and AgNOR sites on individual chromosome pairs.

The distribution pattern of the C-positive band and the AgNOR sites in individual chromosomes is summarized in Tables 2 and 3. The resulting neighbour-joining tree derived from the presence or absence of the characters is shown in Figure 5. The Thrace individuals of *N. leucodon* appeared as the basal branch which was sister to the populations of *N. xanthodon *from Anatolia.

## 4. Discussion

The samples of specimens from each of the populations studied revealed a distinct karyotype which was different from those found in other populations. At the same time, no variation between specimens originating from a single population was recorded, except for the occasionally heterozygous NOR signal observed in some autosomal pairs. The karyotypes found seem identical or similar to those reported by conventional staining in mole rats from the same areas (e.g., [10, 12–16]).

It is difficult to assess exactly the nature of chromosomal variation found between populations without the use of structural banding or chromosome painting. Nevertheless, all the karyotypes examined contained the same number of chromosomes and this uniformity indicates that the Robertsonian changes, proposed as a main mechanism of karyotype divergence in mole rats [13], could hardly participate extensively in the process of chromosomal differentiation among the studied populations. The overall extent of observed variation between the 2*n* = 56 karyotypes from individual geographic populations is rather wide and indicates considerable chromosomal differentiation resulting from varied rearrangements. The role of C-heterochromatin changes and centromeric shifts and inversions can be assumed. The karyotypes with the same diploid number may apparently have evolved separately as a consequence of convergent events.

From the phenetic point of view which can be derived from the banding patterns obtained, the most distinct complement is that of specimens from Thrace. The karyotypes of other populations with 56 chromosomes studied in Anatolia differ from that found in the Thrace individuals in several aspects. The karyotype of the specimens from Thrace includes two large subtelocentric autosomal pairs that obviously do not occur in complements of specimens from the other populations. The large subtelocentric autosomes with the active NOR sites at the short arms were found quite exceptionally in other cytotypes classified as *N. xanthodon* in Anatolia. Similar autosomal pairs were reported only in the 2*n* = 36 and 38 cytotypes from western Anatolia but the short arms were completely C-heterochromatic in the 2*n* = 38 karyotype [22]. On the other hand, similar large subtelocentric autosomal pairs were frequently recorded in karyotypes of the populations classified as *N. leucodon* from southeastern Europe (e.g., [29, 30]).

The number of biarmed autosomes and, consequently, the number of autosomal arms are the highest in the Thrace sample, and also the amount of C-heterochromatin is distinctly larger in this population. In the Anatolian populations, the amount of C-heterochromatin is generally lower, particularly in specimens from the easternmost site in Gülek. The low amount of C-heterochromatin has also been reported in other studied cytotypes of *N. xanthodon *[19–22]. Three large subtelocentric autosomal pairs from the Thrace karyotype possess slight dark C-bands localized in the telomeric region of the short arms and similar C-positive telomeric bands were not recorded in any karyotype of specimens of the Anatolian origin.

The active NOR sites were found on five autosomal pairs in specimens from Thrace and this number was higher than in the other 2*n* = 56 karyotypes where two, three, or four NOR bearing pairs were observed. The lower number of NORs not exceeding three of four pairs was recorded in various cytotypes of *N. xanthodon* in Anatolia (e.g., [19–22, 31]). However, the active NOR sites were recorded in five pairs of subtelocentric autosomes in various populations of the 2*n* = 60 cytotype of *N. xanthodon* from Anatolia [18].

The small size of the X chromosome in the karyotype of specimens from Thrace is quite exceptional among the populations of mole rats studied so far. The X chromosome belonged among the smallest elements in the complement and its relative length was distinctly lower than 6-7% of the haploid set, which is the value usually reported in mammals [32]. The small size of the X chromosome in the Thrace individuals is quite unique. Such a small X chromosome has not been reported from any other *N. leucodon* population in southeastern Europe (e.g., [30]) and it is unknown also in karyotypes of populations belonging to other mole rat species. Moderate variation in the size of the X was recorded also in the 56-chromosome complements of samples studied in Anatolia. The size of the X chromosome in specimens from Yılanlı is only slightly larger than that recorded in the Thrace sample. The X chromosome of the specimens originating Gülek is medium-sized, whereas the X chromosomes of the specimens from Kula and Alaşehir were relatively large.

The large acrocentric autosomes are represented by one or two pairs in the complements of the studied populations. We have not recorded additions of heterochromatic short arms on this chromosome reported in other populations of *N. xanthodon* (e.g., [20]). Surprisingly, we have not found any whole-heterochromatin short arms in the karyotypes examined. The complements of specimens from Kula and Alaşehir in western Anatolia contain two pairs of distinctly large metacentric autosomes. Similar autosomal pairs were found also in the karyotype of the specimens from Yılanlı but the centromere position in the larger pair was submetacentric.

The differences between the studied populations were quantified in the analysis of the distribution pattern of the C-positive bands and the AgNOR sites which indicated a possible divergence pattern with the Thrace individuals of *N. leucodon* appearing as a basal branch of the tree. We are aware that this analysis is only preliminary because of the tentative identification of the individual autosomal pairs; however, the results are congruent with the overall phenetic assessment of the observed chromosomal variation as well as with the results of recent studies of molecular phylogeny [33–35]. The Thrace population, classified as *N. leucodon*, actually appears as a sister group of the Anatolian populations with the same diploid number recognized as *N. xanthodon*.

## 5. Conclusions

We can conclude that the karyotypes of the 56-chromosome populations of *N. leucodon* and *N. xanthodon* are distinctly different from each other but it is difficult to relate directly this difference to considerations of the species status because of the overall extensive karyotypic variation found within the species and the genus. The reproductive isolation, the occurrence of gene flow, and the genetic distance between presumptive species of mole rats should be studied in this respect with the use of molecular approach. Further genetic studies focused on appropriate molecular markers are thus needed to elucidate the problem.

## Figures and Tables

**Table 1:** Studied localities of *Nannospalax leucodon* and *N. xanthodon* in Turkey. The numbering of the sampling sites corresponds to data in Figure 1.

No.	Species	Locality/province	Latitude/longitude	2*n *	No. of specimens		NF	NFa	X	Y
					Male	Female				
1	*N. leucodon *	Babaeski/Kırklareli	41°25′N, 27°07′E	56	1	1	76	72	M	A
2	*N. xanthodon *	Kula/Manisa	38°30′N, 28°36′E	56	2	—	72	68	Sm	A
3	*N. xanthodon *	Alaşehir/Manisa	38°20′N, 28°34′E	56	3	2	74	70	Sm	A
4	*N. xanthodon *	Yılanlı/Isparta	37°47′N, 30°59′E	56	3	—	72	68	Sm	Sm
5	*N. xanthodon *	Gülek/Mersin	37°15′N, 34°45′E	56	3	—	72	68	Sm	A

**Table 2:** The character distribution of the C-positive bands on individual chromosome pairs according to the presence (1) and absence (0). For population numbers see Table 1.

Species/cytotypes	Chromosome no.																												
	1	2	3	4	5	6	7	8	9	10	11	12	13	14	15	16	17	18	19	20	21	22	23	24	25	26	27	X	Y
*N. leucodon*/Babaeski	1	1	1	1	1	1	1	1	1	1	1	1	1	1	1	1	1	1	0	1	1	1	1	1	1	0	0	1	1
*N. xanthodon*/Kula	0	0	0	1	1	1	1	1	1	0	0	0	0	0	0	0	0	0	1	0	0	0	0	0	1	1	0	1	1
*N. xanthodon*/Alaşehir	0	0	0	0	1	1	1	0	1	1	0	0	1	0	0	0	0	0	1	0	0	1	0	0	1	0	0	1	1
*N. xanthodon*/Yılanlı	0	1	1	1	1	1	1	1	0	1	1	1	0	0	1	0	0	0	0	0	0	1	0	0	0	0	0	1	1
*N. xanthodon*/Gülek	0	0	1	1	1	1	1	1	1	0	0	0	0	0	0	0	0	1	0	1	0	0	0	0	0	0	0	1	0

**Table 3:** The character distribution of the AgNOR sites on individual chromosome pairs according to the presence (1) and absence (0). For population numbers see Table 1.

Species/cytotypes	Chromosome no.																												
	1	2	3	4	5	6	7	8	9	10	11	12	13	14	15	16	17	18	19	20	21	22	23	24	25	26	27	X	Y
*N. leucodon*/Babaeski	0	1	1	1	0	1	1	0	0	0	0	0	0	0	0	0	0	0	0	0	0	0	0	0	0	0	0	0	0
*N. xanthodon*/Kula	0	0	0	1	1	0	1	0	0	0	0	0	0	0	0	0	0	0	0	0	0	0	0	0	0	0	0	0	0
*N. xanthodon*/Alaşehir	0	0	0	0	0	1	1	1	0	0	0	0	0	0	0	0	0	0	0	0	0	0	0	0	0	0	0	0	0
*N. xanthodon*/Yılanlı	0	0	0	1	1	1	1	0	0	0	0	0	0	0	0	0	0	0	0	0	0	0	0	0	0	0	0	0	0
*N. xanthodon*/Gülek	0	0	1	0	0	0	0	1	0	0	0	0	0	0	0	0	0	0	0	0	0	0	0	0	0	0	0	0	0

## References

[1] Musser G, Carleton M, Wilson DE, Reeder DM (2005). Superfamily Muroidea. *Mammal Species of the World. A Taxonomic and Geographic Reference*.

[2] Nevo E, Reig AO (1990). *Evolution of Subterranean Mammals at the Organismal and Molecular Levels*.

[3] Begall S, Burda H, Schleich CE (2007). *Subterranean Rodents. News from Underground*.

[4] Nevo E, Ivanitskaya E, Beiles A (2001). *Adaptive Radiation of Blind Subterranean Mole Rats: Naming and Revisiting the Four Sibling Species of the Spalax ehrenbergi superspecies in Israel: Spalax galili (2n=52), S. golani (2n=54), S. carmeli (2n=58) and Spalax judaei (2n=60)*.

[5] Chişamera G, Bužan EV, Sahlean T, Murariu D, Zupan S, Kryštufek B (2014). Bukovina blind mole rat *Spalax graecus* revisited: phylogenetics, morphology, taxonomy, habitat associations and conservation. *Mammal Review*.

[6] Yiğit N, Çolak E, Sözen M, Karataş A (2006). *Rodents of Türkiye*.

[7] Kryštufek B, Vohralík V (2009). *Mammals of Turkey and Cyprus. Rodentia II: Cricetinae, Muridae, Spalacidae, Calomyscidae, Capromyidae, Hystricidae, Castoridae*.

[8] Nevo E, Filippucci MG, Redi C, Simson S, Heth G, Beiles A (1995). Karyotype and genetic evolution in speciation of subterranean mole rats of the genus *Spalax* in Turkey. *Biological Journal of the Linnean Society*.

[9] Sözen M, Çolak E, Yiğit N (2000). A study on karyotypic evolution of the genus *Spalax* Güldenstaedt, 1770 (Mammalia: Rodentia) in Turkey. *Israel Journal of Zoology*.

[10] Sözen M, Matur F, Çolak E, Özkurt Ş, Karataş A (2006). Some karyological records and a new chromosomal form for *Spalax* (Mammalia: Rodentia) in Turkey. *Folia Zoologica*.

[11] Savić I, Soldatović B (1979). Contribution to the knowledge of the genus *Spalax (Microspalax)* karyotype from Asia Minor. *Archives of Biological Sciences*.

[12] Sözen M (2004). A karyological study on subterranean mole rats of the *Spalax leucodon* Nordmann, 1840 superspecies in Turkey. *Mammalian Biology*.

[13] Matur F, Çolak F, Ceylan T, Sevindik M, Sözen M (2013). Chromosomal evolution of the genus *Nannospalax* (Palmer 1903) (Rodentia, Muridae) from western Turkey. *Turkish Journal of Zoology*.

[14] Kankılıç T, Kankılıç T, Seker PS, Çolak R, Selvi E, Çolak E (2010). Contributions to the karyology and distribution areas of cytotypes of *Nannospalax leucodon* (Rodentia: Spalacidae) in Western Anatolia. *Acta Zoologica Bulgarica*.

[15] Sözen M, Çatakli K, Eroğlu F, Matur F, Sevindik M (2011). Distribution of chromosomal forms of *Nannospalax nehringi* (Satunin, 1898) (Rodentia: Spalacidae) in Çankiri and Çorum provinces, Turkey. *Turkish Journal of Zoology*.

[16] Sözen M, Çolak F, Sevindik M, Matur F (2013). Cytotypes of *Nannospalax xanthodon* (Satunin, 1898) (Rodentia, Spalacidae) from western Anatolia. *Turkish Journal of Zoology*.

[17] Ivanitskaya E, Coskun Y, Nevo E (1997). Banded karyotypes of mole rats (*Spalax*, Spalacidae, Rodentia) from Turkey: a comparative analysis. *Journal of Zoological Systematics and Evolutionary Research*.

[18] Ivanitskaya E, Sözen M, Rashkovetsky L, Matur F, Nevo E (2008). Discrimination of 2n=60 *Spalax leucodon* cytotypes (Spalacidae, Rodentia) in Turkey by means of classical and molecular cytogenetic techniques. *Cytogenetic and Genome Research*.

[19] Arslan A, Bölükbaş F (2010). C-heterochromatin and NORs distribution of mole rat, *Nannospalax xanthodon* from Aksaray, Turkey. *Caryologia*.

[20] Arslan A, Akan Ş, Zima J (2011). Variation in C-heterochromatin and NOR distribution among chromosomal races of mole rats (Spalacidae) from Central Anatolia, Turkey. *Mammalian Biology*.

[21] Arslan A, Zima J (2013). The banded karyotype of the 2n=58 chromosomal race of mole rats from Erzincan, Turkey. *Folia Zoologica*.

[22] Arslan A, Arısoy A, Zima J (2013). The chromosome banding pattern in two cytotypes (2n=36 and 38) of blind mole rats from Turkey (Mammalia: Spalacidae). *Zoology in the Middle East*.

[23] Arslan A (2013). A new live trap to catch blind mole rats (*Spalax* sp.). *Folia Zoologica*.

[24] Ford CE, Hamerton JL (1956). A colchicine, hypotonic citrate, squash sequence for mammalian chromosomes. *Stain Technology*.

[25] Sumner AT (1972). A simple technique for demonstrating centromeric heterochromatin. *Experimental Cell Research*.

[26] Howell WM, Black DA (1980). Controlled silver-staining of nucleolus organizer regions with a protective colloidal developer: a 1-step method. *Experientia*.

[27] Hsu TC, Benirschke K (1967–1977). *An Atlas of Mammalian Chromosomes, Vol. 1–10*.

[28] Hammer Ø, Harper DAT, Ryan PD (2001). Past: paleontological statistics software package for education and data analysis. *Palaeontologia Electronica*.

[29] Savić I, Soldatović B (1979). Distribution range and evolution of chromosomal forms in the Spalacidae of the Balkan Peninsula and bordering regions. *Journal of Biogeography*.

[30] Savić I, Soldatović B (1984). Karyotype evolution of the genus Nannospalax Palmer, 1903, Mammalia, in Europe. *Serbian Academy of Sciences and Art, Separate Edition*.

[31] Gülkaç MD, Küçükdumlu İ (1999). Variation in the nucleolus organizer regions (NORs) in two mole rat species (*Spalax leucodon* and *Spalax ehrenbergi*). *Turkish Journal of Biology*.

[32] Ohno S (1969). Evolution of sex chromosomes in mammals. *Annual Review of Genetics*.

[33] Arslan E, Gülbahçe E, Arikoğlu H, Arslan A, Bužan EV, Kryštufek B (2010). Mitochondrial divergence between three cytotypes of the Anatolian mole rat *Nannospalax xanthodon* (Mammalia: Rodentia). *Zoology in the Middle East*.

[34] Kandemir I, Sözen M, Matur F (2012). Phylogeny of species and cytotypes of mole rats (Spalacidae) in Turkey inferred from mitochondrial cytochrome *b* sequences. *Folia Zoologica*.

[35] Kryštufek B, Ivanitskaya E, Arslan A, Arslan E, Bužan EV (2012). Evolutionary history of mole rats (genus *Nannospalax*) inferred from mitochondrial cytochrome *b* sequence. *Biological Journal of the Linnean Society*.

